# Mastectomy and Immediate Breast Reconstruction with Pre-Pectoral or Sub-Pectoral Implant: Assessing Clinical Practice, Post-Surgical Outcomes, Patient’s Satisfaction and Cost

**DOI:** 10.26502/jsr.10020250

**Published:** 2022-09-09

**Authors:** Gilles Houvenaeghel, Monique Cohen, Laura Sabiani, Aurore Van Troy, Olivia Quilichini, Axelle Charavil, Max Buttarelli, Sandrine Rua, Agnès Tallet, Alexandre de Nonneville, Marie Bannier

**Affiliations:** 1 Aix-Marseille University, CNRS (National Center of Scientific Research), INSERM (National Institute of Health and Medical Research), Paoli-Calmettes Institute, Department of Surgical Oncology, CRCM (Research Cancer Centre of Marseille), 13009 Marseille, France; 2 Paoli-Calmettes Institute, Department of Surgical Oncology, CRCM (Research Cancer Centre of Marseille), 13009 Marseille, France; 3 Paoli-Calmettes Institute, Department of Radiotherapy, CRCM (Research Cancer Centre of Marseille), 13009 Marseille, France; 4 Aix-Marseille University, CNRS (National Center of Scientific Research), INSERM (National Institute of Health and Medical Research), Paoli-Calmettes Institute, Department of Medical Oncology, CRCM (Research Cancer Centre of Marseille), 13009 Marseille, France

**Keywords:** Breast cancer, Clinical practice, reconstruction, pre-pectoral implant, post-surgical outcome

## Abstract

Immediate breast reconstruction (IBR) rates increase during last years and implant-based reconstruction was the most commonly performed procedure. We examined data collected over 25 months to assess complication rate, duration of surgery, patient’s satisfaction and cost, according to pre-pectoral or sub-pectoral implant-IBR.

All patients who received an implant-IBR, from January 2020 to January 2022, were included. Results were compared between pre-pectoral and sub-pectoral implant-IBR in univariate and multivariate analysis.

We performed 316 implant-IBR, 218 sub-pectoral and 98 (31%) pre-pectoral. Pre-pectoral implant-IBR was significantly associated with the year (2021: OR=12.08 and 2022: OR=76.6), the surgeons and type of mastectomy (SSM vs NSM: OR=0.377).

Complications and complications Grade 2–3 rates were 12.9% and 10.1% for sub-pectoral implant-IBR respectively, without significant difference with pre-pectoral implant-IBR: 17.3% and 13.2%. Complications Grade 2–3 were significantly associated with age <50-years (OR=2.27), ASA-2 status (OR=3.63) and cup-size >C (OR=3.08), without difference between pre and sub-pectoral implant-IBR. Durations of surgery were significantly associated with cup-size C and >C (OR=1.72 and 2.80), with sentinel lymph-node biopsy and axillary dissection (OR=3.66 and 9.59) and with sub-pectoral implant-IBR (OR=2.088). Median hospitalization stay was 1 day, without difference between pre and sub-pectoral implant-IBR. Cost of surgery was significantly associated with cup-size > C (OR=2.216) and pre-pectoral implant-IBR (OR=8.02). Bad-medium satisfaction and IBR-failure were significantly associated with local recurrence (OR=8.820), post-mastectomy radiotherapy (OR=1.904) and sub-pectoral implant-IBR (OR=2.098).

**Conclusion:**

Complications were not different between pre and sub-pectoral implant-IBR. Pre-pectoral implant-IBR seems a reliable and faster technique with better patient satisfaction but with higher cost.

## 2. Introduction

Total mastectomies for breast cancer (BC) are still indicated for 12 to 30% of patients and up to 40% [1–5]. It was 12.2% in a large French cohort of invasive BC and mastectomy rate increased according to four successive periods from 6.1% to 23.2% [6]. For risk reducing mastectomy, unilateral or bilateral mastectomies are indicated for BRCA mutations and for patients without BRCA mutation with estimated-risk of BC up to 25%. Immediate breast reconstruction (IBR) rates increase during the last years [7] in order to improve quality of life [8] and implant-based reconstruction was the most commonly performed procedure [9–11]. In our center, among 2112 mastectomies performed between January 2016 and July 2020, IBR-rate was 40.5%: 35.4% (618/1748) for primary BC, 47.9% (105/219) for local recurrence and 91% (132/145) for prophylactic mastectomies. Several new procedures are been developed, as robotic procedures [12–15], pre-pectoral implant-IBR with or without synthetic or acellular dermal matrix [7,16–21]. However, it was reported that use of meshes significantly increases the cost of surgery [16]. Moreover, in recent year’s nipple sparing mastectomy (NSM) is more and more frequently performed for prophylactic mastectomies [17], for local recurrence [18] and for primary BC [19,20]. Generally, the NSM studies reported better aesthetic results than skin-sparing mastectomy (SSM) and better quality of life [22–24]. NSM with IBR is consider today as a valid procedure for prophylactic mastectomy [17,25–28] and an acceptable option for BC therapeutic mastectomy [29–31]. Complication rates varies between 5% and 61% in literature [32]. This wide difference in complications rates is explained by the difficulty of comparing the results of different studies due to the large disparities in IBR rates and techniques, the complications reported, the indications for mastectomies and the monitoring time. However, increased body mass index (BMI) and smoking were reported factors to increase the risk of complications as well as previous radiotherapy and operative time [21]. In this study, we report our experience at the Paoli Calmettes Institute, by analyzing the data collected over 25 months from January 2020 to January 2022 to assess clinical practice, complication rate, duration of surgery, patient’s satisfaction and cost, according to pre-pectoral or sub-pectoral implant-IBR.

## Methods

All patients who received an implant-IBR, from January 2020 to January 2022, were included, regardless of the indication for mastectomy from institutional database (study: M-IBR-PPRP-IPC 2022–014). The main characteristics were collected prospectively: year of IBR, use of matrix, type of mastectomy (NSM, SSM, or standard) and indication, associated axillary procedure, neo-adjuvant chemotherapy, age, duration of surgery, mastectomy weight, implant size, history of radiotherapy, ASA status (American Society of Anesthesiologists), smoking status, BMI and surgeon. Analyses were realized on all mastectomies, including two mastectomies for patients with bilateral procedures. Duration of anesthesia for bilateral mastectomies were halved. These criteria were compared between the two pre-pectoral and sub-pectoral prosthesis groups in univariate analysis and logistic regression. The following were analyzed: factors associated with the indication of IBR type, complications appeared in 90 days following the operation and grade 2–3 complications (Clavien Dindo classification), duration of surgery, and type of incision. The operative time was recorded from skin incision to skin closure. The choice between the two techniques was made by the surgeon and the matrices used were resorbable synthetic mesh TIGR Matrix^®^ (Novus Scientific, Uppsala, Sweden). The length of postoperative stay (LPOS) was reported from the surgery day to the discharge day from hospital. A loco regional anesthesia with pectoralis block was systematically perform. Costs of initial procedures were evaluated with costs addition of implant (400 Euros), number of hospitalization days (1495.69 Euros per day), duration of anesthesia (402.54 Euros per hour) and matrix TIGR (1390 Euros for 20×30 centimeters). Patient’s satisfaction were evaluated as bad, medium, good and very good for patients without re-operation with implant loss for complication.

### Statistics

Quantitative criteria were analyze with median, mean, 95% CI. Comparisons were determined using the Chi-2 test for qualitative criteria and t-test for quantitative criteria. Factors significantly associated with criteria analyzed were determine by a binary logistic regression adjusted for all significant variables identified by the univariate analysis. An odds ratio (OR) with a 95% confidence interval (95% CI) was used as an effective measure. Statistical significance was assessed at p < 0.05. Analyses were perform with SPSS version 16.0 (SPSS Inc., Chicago, Illinois).

## Results

### Population

During the 25-month period, 316 implant-IBR, 218 IBR by sub-pectoral prosthesis and 98 (31%) IBR by pre-pectoral prosthesis (85 times associated with a matrix: 97.7%) were performed. Forty height bilateral mastectomies were performed in 24 patients: 17 for primary BC and 31 prophylactic (64.6%). During the same period, 904 mastectomies were performed, including 348 IBR (38.5%): 316 implant-IBR, 20 expander first IBR, 12 latissimus dorsi-flap IBR.

### Pre or sub pectoral implant-IBR

Characteristics of patients according to pre or sub pectoral implant-IBR are reported in table 1. Bilateral mastectomy was performed in 48 patients: 29 in sub-pectoral group and 19 in pre-pectoral group. In univariate analysis, pre-pectoral implant IBR was significantly associated with years, NSM, axillary surgery type, neo-adjuvant chemotherapy, surgeon and age. There was no significant difference for mastectomy weight, histology, indication of mastectomy, breast size, smokers, ASA status, previous homo-lateral breast surgery, radiotherapy.

Pre or sub pectoral implant-IBR rates according to year of surgery and surgeons are reported in table 2. A great increase of pre-pectoral implant-IBR rate was observed for the four surgeons who realized pre-pectoral implant-IBR.

An IBR by pre-pectoral prosthesis was significantly associated with the year (2021: OR=12.08 and 2022: OR=76.6), the surgeons and the type of mastectomy (SSM vs NSM: OR=0.377) (Table 3).

Incisions used for NSM were localized in breast inferior fold in the majority of patients (91%: 61/67) (Table 4).

### Complications

Complication rates and complications Grade 2–3 rates were 12.9% (28/217) and 10.1% (22/217) for sub-pectoral implant-IBR respectively, without significant difference with pre-pectoral implant-IBR: 17.3% (17/98) and 13.2% (13/98), respectively (Table 5).

Complication type according to pre or sub-pectoral implant-IBR are reported in table 6, there was no significant difference (p=0.301). Implant loss rates were not significantly different: 6.40% and 9.20% for retro and pre-pectoral implant-IBR, respectively.

In multivariate analysis, complications were significantly associated with age <50 years (OR=2.0) and complications Grade 2–3 were significantly associated with age <50 years (OR=2.27) and ASA 2 status (OR=3.63) and breast cup-size >C (OR=3.08), without difference between pre and sub pectoral implant-IBR (Table 7).

### Duration of surgery

Median duration of surgery was 90.5 minutes (mean 97.1, CI95% 93.7–100.5), significantly higher for sub-pectoral implant-IBR in comparison with pre-pectoral implant-IBR: 100 minutes versus 80 minutes, p<0.0001. In multivariate analysis, medians duration of surgery higher than median value were significantly associated with breast cup size C and >C (OR=1.72 and 2.80, respectively), with sentinel lymph node biopsy and axillary lymph node dissection (OR=3.66 and 9.59, respectively), and with sub-pectoral implant-IBR (OR=2.088) in comparison with pre-pectoral implant-IBR (Table 8).

### Length of postoperative stay (LPOS)

Median LPOS was 1 day (mean: 1.47, SE: 0.042, CI95%: 1.39–1.55, range: 1–5), without significant difference between pre and sub pectoral implant-IBR (p=0.090, mean: 1.39 and 1.51 respectively). A significant difference was observed between implant size <= versus > 300gr (p=0.001): mean, 1.42 versus 1.97 days, respectively.

### Adjuvant therapy

Neo-adjuvant chemotherapy (NAC) was delivered in 46 patients: for 23 patients among 136 patients with invasive BC (16.9%) in sub-pectoral implant-IBR group and for 23 patients among 71 patients with invasive BC (32.4%) in pre-pectoral implant-IBR group. Adjuvant chemotherapy was delivered in 59 patients among 150 patients with invasive BC without NAC (39.3%): for 39 patients among 112 patients with invasive BC without NAC (34.8%) in sub-pectoral implant-IBR group and for 20 patients among 48 patients with invasive BC without NAC (41.7%) in pre-pectoral implant-IBR group (Table 1). Post mastectomy radiotherapy (PMRT) was delivered in 61 patients among 194 patients with invasive BC without previous radiotherapy (31.4%): for 40 patients among 123 patients with invasive BC without previous radiotherapy (32.5%) in sub-pectoral implant-IBR group and for 21 patients among 71 patients with invasive BC without previous radiotherapy (29.6%) in pre-pectoral implant-IBR group.

### Cost evaluation

Initial surgery cost: Median cost for all patients was 3981.8 Euros (mean 3949.1, CI95% 3813–4085): 3174 (3668, 3514–3821) for sub-pectoral implant-IBR and 4228 (4575, 4341–4809) for pre-pectoral implant-IBR (p<0.0001) with a median difference of 1054 Euros between two groups. In multivariate analysis, cost of surgery higher than median value was significantly associated with breast cup-size > C (OR=2.216, CI95% 1.04–4.71, p=0.039) and pre-pectoral implant-IBR (OR=8.02, CI95% 4.43–14.55, p<0.0001). Axillary surgery and breast cup-size C were non-significant.

### Satisfaction

Patient’s satisfaction, evaluated before re-operation for lipofilling or change of breast implant, is reported in table 9. When satisfactions results were classified in two categories, good and very good versus bad, medium and failure, several significant factors were observed: pre-pectoral versus sub-pectoral implant-IBR (p=0.035), indication of mastectomy (p<0.0001) and radiotherapy (p=0.020). In binary logistic regression, bad, medium and IBR-failure were significantly associated with mastectomy for local recurrence (OR=8.820, CI95% 2.63–29.56, p<0.0001), with PMRT (OR=1.904, CI95% 1.03–3.52, p=0.040) and sub-pectoral implant-IBR (OR=2.098, CI95% 1.18–3.74, p=0.012).

## Discussion

We report in this retrospective monocentric study an important number of patients with implant-IBR, during a recent and short period, with a high rate of IBR. Pre-pectoral implant-IBR were performed significantly more frequently for NSM. In multivariate analysis, complications Grade 2–3 were significantly associated with age <50 years, ASA 2 status and breast cup-size >C, without difference between pre and sub pectoral implant-IBR. Shorter duration of surgery was reported for pre-pectoral implant-IBR. A high rate of IBR reflects a relatively poor selection of patients for whom reconstruction is proposed. Conversely, a low rate of IBR is most likely related to a large selection of patients in whom an IBR is proposed. This can induce evaluation biases, particularly on complications rate, the patients most at risk having been excluded. In our practice, an IBR has been very widely proposed by excluding patients at very high risk of complication, or patients whose choice was not to perform an IBR. Patients considered to be at very high risk correspond to patients with significant sequelae from previous radiotherapy, or patients with significant and/or multiple co-morbidities. Inflammatory BC was also excluded to IBR. In this study, IBR rate was 38.5% which is relatively high and much higher than reported in the literature. Despite the COVID-19 pandemic, and a decrease in surgeries during this period having included several outbreaks, the number of surgeries for BC and the IBR rate were high [33,34]. The IBR rate in France was therefore assess at 16.1% in an observational study between 2008 and 2014 among 140,904 women who had undergone a total mastectomy for BC [7]. In England, the number of implant-IBR have increased since 2009: 10.0% until 2005 and 23.3% by 2013–2014 [9]. In Chinese, IBR rate was 9.6% (1,554/16,187) in year 2018, with implant or expander in 76.6% of these IBR [11]. However, the average rate of reconstruction in the United States in 2010 was 45%, surging to 54% in 2015 [35]. In the UK multicenter prospective cohort study [21], 2108 patients had 2655 mastectomies with implant-IBR in 81 units during 28 months: 11 patients’ per-year per unit in comparison with 152 implant-IBR patient’s per-year in this study. Moreover, Wow et al. [36] recently reported 232 implant-IBR with definitive implant or expander in two centers during 31 months (45 patients per-year per-center) including 123 risk-reducing mastectomy (53.0%) and a low rate of implant-IBR for BC (109 patients: 47%). In our study 85.8% of implant-IBR was performed for BC. IBR and NSM is possible for patients with ipsilateral local recurrence after initial conservative surgery with radiotherapy for BC in selective cases as we reported [18]. Pre-pectoral implant-IBR significantly increase according to years of treatment (6.0% to 72.7%) similarly to results reported by King et al. [37] (0% to 92.4%). Implant-IBR rate and type of mastectomy: pre-pectoral implant-IBR was performed less frequently for SSM than NSM, in Wow et al., study [36] (10.6% versus 81.6%, respectively) as we report (18.4% versus 45.9%). It is difficult to compared absolute complications rates between studies, due to a large disparity of IBR types, reported complications, indications for mastectomies, and monitoring time. Complication rates with pre-pectoral versus sub-pectoral implant-IBR were similar in meta-analysis reported by Li et al [38] and in meta-analysis reported by Chatterjee et al [39], but with lower odds of infection for pre-pectoral implant-IBR, and with higher rate of smokers, PMRT and diabetes in sub-pectoral procedures. Minor complications occurred more often for sub-pectoral procedures in Wow et al [36] study (26.32% versus 5.77%). In contrast, we don’t report difference (2.76% versus 4.08%) like Momeni et al [40] (30% for pre-pectoral and 22.5% for sub-pectoral). We don’t observe difference of major complications rates between pre and sub-pectoral implant-IBR like others, 10.9% pre versus 9.21% sub pectoral [36]. However, Momeni et al [40] reported higher major complication rate for sub-pectoral procedure (22.5% versus 7.5%) without significant difference (p=0.060). Significant higher prosthetic failure rate was reported for sub-pectoral versus pre-pectoral in King et al study [37] (18.7% versus 7.9%), but without significant difference in Momeni et al study [40] (2.5 versus 12.5% for pre and sub-pectoral implant IBR respectively), in meta-analysis reported by Chatterjee [39] and meta-analysis reported by Li et al [38], and without difference in the present study. A significant shorter median hospitalization time for pre-pectoral implant-IBR was reported by Wow et al [36], with a median time of 4 days for all patients. With a shorter median LPOS of 1 day, we don’t observe difference between pre and sub-pectoral implant-IBR. Consequently, despite a shorter duration of anesthesia for pre-pectoral implant-IBR, we reported higher cost for pre-pectoral implant-IBR in comparison with sub-pectoral implant-IBR, as reported by Chopra et al [16]. Significant lower postoperative pain have been reported in pre-pectoral implant-IBR whereas others reported that the pain scores were not significantly different, without conclusion in Li et al meta-analysis [38]. Moreover, there was no significance difference of quality of life between the pre-pectoral and sub-pectoral groups in Li et al meta-analysis [38].

Several limitations of this study can be underlined: 1) retrospective design, even if the data was collected prospectively, 2) mono-centric study, 3) cost evaluation of initial surgery without true medico-economic study, 4) patient’s satisfaction: patient satisfaction remains subjective but represents the predominant evaluation factor in relation to the opinion of doctors.

## Conclusion

Complications Grade 2–3 were significantly associated with age <50 years, ASA 2 status and breast cup-size >C, without difference between pre and sub pectoral implant-IBR. Despite a shorter duration of surgery, higher cost was observed for pre-pectoral implant-IBR. More patients achieved bad or medium satisfaction for local recurrence, with PMRT and for sub-pectoral implant-IBR. Pre-pectoral implant-IBR seems to correspond to a reliable, faster technique with equivalent results in terms of complications and better patient satisfaction. To confirm these results, a multicenter study is ongoing.

## Figures and Tables

**Table 1:** Characteristics of patients according to pre or sub pectoral implant-IBR

		Sub Pectoral		Pre Pectoral		Chi^2^
		Nb	%	Nb	%	*p*
		218	69	98	31	
Mesh	No	216	94.3	13	5.7	**<0,0001**
	Yes	2	2.3	85	97.7	
Years	2020	109	94	7	6	**<0,0001**
	2021	103	57.9	75	42.1	
	2022	6	27.3	16	72.7	
Mastectomy type	NSM	79	54.1	67	45.9	**<0,0001**
	SSM	137	81.5	31	18.5	
	Standard	2	100	0	0	
Axillary surgery	No	83	61.5	52	38.5	**0.022**
	SLNB	116	76.3	36	23.7	
	ALND	18	64.3	10	35.7	
NAC	No	194	72.1	75	27.9	**0.005**
	Yes	23	50	23	50	
Adjuvant	No	73	72.3	28	27.7	0.4
Chemotherapy*	Yes	39	66.1	20	33.9	
Surgeons	1	18	32.1	38	67.9	**<0,0001**
	2	32	100	0	0	
	3	50	75.8	16	24.2	
	4	18	100	0	0	
	5	44	52.4	40	47.6	
	6	20	83.3	4	16.7	
	7	29	100	0	0	
	8	7	100	0	0	
Histology	DCIS	49	77.8	14	22.2	0.196
	NST	111	67.3	54	32.7	
	Lobular	25	61	16	39	
	Others	0	0	1	100	
	Begnin	32	71.1	13	28.9	
Indication	Primary	166	69.7	72	30.3	0.62
	Recurrence	19	61.3	12	38.7	
	Prophylactic	33	70.2	14	29.8	
Cup-size	A-B	115	68.5	53	31.5	0.9
	C	71	70.3	30	29.7	
	> C	30	66.7	15	33.3	
Radiotherapy	No	162	67.8	77	32.2	0.098
	PMRT	40	65.6	21	34.4	
	previous RTH	12	100	0	0	
	NAC + N-RTH	1	100	0	0	
Previous homolateral	No	137	67.2	67	32.8	0.368
surgery	Yes	80	72.1	31	27.9	
Smoker	No	175	68.4	81	31.6	0.672
	Yes	42	71.2	17	28.8	
ASA-status	1	102	72.3	39	27.7	0.39
	2	111	65.7	58	34.3	
	3	4	80	1	20	
Bilateral	No	189	70.5	79	29.5	0.162
	Yes	29	60.4	19	39.6	

* adjuvant chemotherapy for invasive breast cancer without neo-adjuvant chemotherapy

Significant values: in bold characters

Abbreviations: NSM, nipple sparing mastectomy; SSM, skin sparing mastectomy; SLNB, sentinel lymph node biopsy; ALND, axillary lymph node dissection; ASA, American Society of Anesthesiologists; BMI, body mass index; RTH, radiotherapy; BC, breast cancer; PMRT, post-mastectomy radiotherapy; NAC, neo-adjuvant chemotherapy; N-RTH, neo-adjuvant radiotherapy; DCIS, Ductal carcinoma in-situ; NST, Nonspecific tumor (ductal invasive)

**Table 2:** Pre-pectoral implant-IBR according to surgeons and year of surgery.

**% Pre pectoral**	**Years**	**2020**		**2021**		**2022**	
Surgeon		**Nb**	**%**	**Nb**	**%**	**Nb**	**%**
	1	(1/14)	7.1	(33/38)	86.8	(4/4)	100
	3	(5/28)	17.9	(6/32)	19.8	(5/7)	71.4
	5	(1/22)	4.5	(34/57)	59.6	(5/5)	100
	6	(0/6)	0	(2/14)	14.3	(2/4)	50
	2-4-7-8	(0/47)	0	(0/37)	0	(0/1)	0

**Table 3:** Pre-pectoral versus sub-pectoral implant-IBR- regression analysis.

Regression indication Pre versus Sub pectoral		*p*	Odd Ratio	CI 95.0%	
				Inferior	Superior
Year	2020	<0.0001	1		
	2021	**<0.0001**	**12.084**	4.672	31.254
	2022	**<0.0001**	**76.641**	15.021	391.04
Surgeons	1	<0.0001	1		
	2	0.997	0	0	.
	3	**<0.0001**	**0.109**	0.04	0.299
	4	0.998	0	0	.
	5	**0.009**	**0.3**	0.122	0.74
	6	**<0.0001**	**0.037**	0.008	0.167
	7	0.997	0	0	.
	8	0.999	0	0	.
Mastectomy type	NSM	0.019	1		
	SSM	**0.005**	**0.377**	0.191	0.744
	Standard	0.999	0	0	.
Age	<50 vs >=50	0.584	0.82	0.402	1.669
Implant size	> vs <= 300	0.464	0.765	0.374	1.566

Abbreviations: NSM, nipple sparing mastectomy; SSM, skin sparing mastectomy.

**Table 4:** Incisions for NSM according to Pre or sub pectoral implant-IBR.

NSM incisions	Sub-pectoral		Pre-pectoral		Chi^2^
	Nb	%	Nb	%	p
	80	54.4	67	45.6	
axillar	4	5	4	6	<0.0001
areolar	1	1.2	0	0	
central	2	2.5	0	0	
inversed T	3	3.8	0	0	
areolar and radial	19	23.8	0	0	
radial	6	7.5	2	3	
inferior fold	45	56.2	61	91	

Abbreviations: NSM, nipple sparing mastectomy.

**Table 5:** Complications according to pre or sub pectoral implant-IBR.

		Sub-pectoral		Pre-pectoral		Chi^2^
		Nb	%	Nb	%	p
Complication	No	189	70	81	30	0.301
	Yes	28	62.2	17	37.8	
Grade 2–3 Complication	No	195	69.6	85	30.4	0.441
	Yes	22	62.9	13	37.1	
Implant loss		14	6.4	9	9.2	0.382
Re operation	No	199	70.1	85	29.9	0.149
	Yes	17	56.7	13	43.3	

**Table 6:** Complication type according to pre or sub-pectoral implant-IBR.

Complication type		Sub-pectoral	Pre-pectoral	Total
cutaneous / NAC	Nb	14	3	17
	%	0.5	0.25	0.425
hematoma	Nb	8	7	15
	%	0.286	0.583	0.375
infection	Nb	5	2	7
	%	0.179	0.167	0.175
others	Nb	1	0	1
	%	0.036	0	0.025

Abbreviations: NAC, nipple areolar complex.

**Table 7:** Complication all grades and complications grade 2–3, in multivariate analysis.

**Complications all Grades**		**p**	**Odd Ratio**	**CI 95.0%**	
				**Inferior**	**Superior**
Mastectomy	NSM	0.104	1		
	SSM	0.078	0.526	0.258	1.073
	Standard	0.321	5.155	0.202	131.72
**Age**	<50 vs >=50	**0.06**	**2.002**	0.972	4.122
Implant size	> vs <= 300	0.19	1.614	0.789	3.302
Implant	Pre vs Sub Pectoral	0.664	1.172	0.572	2.401
Smoker	yes vs no	0.415	1.419	0.612	3.288
ASA	1	0.266	1		
	2	0.107	1.848	0.875	3.902
	3	0.547	2.041	0.2	20.785
BMI	<=24.99	0.693	1		
	25–29.99	0.723	1.164	0.502	2.696
	>=30	0.405	2.167	0.35	13.403
**Complications Grade 2–3**		**p**	**Odd Ratio**	**CI 95.0%**	
				**Inferior**	**Superior**
Mastectomy	NSM	0.17	1		
	SSM	0.342	0.679	0.305	1.51
	Standard	0.132	12.909	0.463	359.77
**Age**	<50 vs >=50	**0.06**	**2.273**	0.967	5.342
Implant size	<= vs > 300	0.757	1.148	0.479	2.752
**ASA**	1	0.032	1		
	2	**0.009**	**3.63**	1.386	9.511
	3	0.999	0	0	.
Implant	Pre vs Sub Pectoral	0.672	1.193	0.527	2.7
previous RTH	yes vs no	0.332	1.65	0.6	4.535
**Cup-size**	A-B	0.056	1		
	C	0.947	0.968	0.375	2.499
	**>C**	**0.037**	**3.082**	1.07	8.881

Abbreviations: NSM, nipple sparing mastectomy; SSM, skin sparing mastectomy; ASA, American Society of Anesthesiologists; BMI, body mass index; RTH, radiotherapy.

**Table 8:** Medians duration of surgery higher than median value in multivariate analysis.

Duration of surgery		*p*	Odd Ratio	CI 95.0%	
Regression analysis				Inferior	Superior
Cup size	A-B	0.012	1		
	C	**0.05**	**1.721**	1	2.963
	> C	**0.007**	**2.799**	1.321	5.929
Axillary	No	**<0.0001**	1		
surgery	SLNB	**<0.0001**	**3.663**	2.191	6.122
	ALND	**<0.0001**	**9.594**	3.537	26.02
Implant	Sub vs Pre	**0.007**	**2.088**	1.218	3.579

Abbreviations: SLNB, sentinel lymph node biopsy; ALND, axillary lymph node biopsy

**Table 9:** Patient’s satisfaction according to characteristics of patients and surgery.

Satisfaction		implant loss		bad		medium		good		very good		Chi^2^	Chi^2^*
		Nb	%	Nb	%	Nb	%	Nb	%	Nb	%	*p*	*p*
Implant-IBR	Pre-pectoral	9	9.2	8	3.1	13	13.3	49	50	24	24.5	0.076	0.035
	Sub-pectoral	15	6.9	3	3.7	59	27.1	99	45.4	37	17		
BMI	< 25	17	6.9	8	3.3	63	25.6	114	46.3	44	17.9	0.09	0.403
	25–29.9	5	7.9	3	4.8	9	14.3	29	46	17	27		
	>= 30	2	28.6	0	0	0	0	5	71.4	0	0		
ASA	1	4	2.8	5	3.5	30	21.3	72	51.1	30	21.3	0.077	0.067
	2	20	11.8	6	3.5	39	22.9	74	43.5	31	18.2		
	3	0	0	0	0	3	60	2	40	0	0		
Implant size	<= 300	8	4.5	9	5.1	40	22.7	84	47.7	35	19.9	0.088	0.562
	> 300	16	11.6	2	1.4	31	22.5	63	45.7	26	18.8		
Cup-size	A-B	10	6	4	2.4	39	23.2	84	50	31	18.5	0.485	0.355
	> B	14	9.5	7	4.7	33	22.3	64	43.2	30	20.3		
Age	<= 40	2	2.7	2	2.7	18	24.3	32	43.2	20	27	0.066	0.11
	41–50	3	3.2	3	3.2	19	20.2	48	51.1	21	22.3		
	51–74	18	13.2	6	4.4	32	23.5	60	44.1	20	14.7		
	>= 75	1	8.3	0	0	3	25	8	66.7	0	0		
Type mastectomy	NSM	14	9.5	5	3.4	31	21.1	63	42.9	34	23.1	0.216	0.886
	SSM	9	5.4	6	3.6	41	24.6	84	50.3	27	16.2		
	Standard	1	50	0	0	0	0	1	50	0	0		
Indication	Primary BC	14	5.9	8	3.4	55	23.2	113	47.7	47	19.8	0.001	<0.0001
	Local recurrence	8	25.8	1	3.2	11	35.5	9	29	2	6.5		
	Prophylactic	2	4.2	2	4.2	6	12.5	26	54.2	12	25		
Smoker	No	18	7	9	3.5	58	22.5	130	50.4	43	16.7	0.047	0.468
	Yes	6	10.3	2	3.4	14	24.1	18	31	18	31		
Radiotherapy	No	15	6.2	8	3.3	50	20.7	119	49.2	50	20.7	0.372	0.02
	PMRT	7	11.5	3	4.9	16	26.2	25	41	10	16.4		
	previous RTH	2	16.7	0	0	6	50	3	25	1	8.3		
	NAC + N-RTH	0	0	0	0	0	0	1	100	0	0		
Total		24	7.6	11	3.5	72	22.9	147	46.7	61	19.4		

* Chi^2^ (good and very good satisfaction) versus (failure-bad-medium).

Abbreviations: NSM, nipple sparing mastectomy; SSM, skin sparing mastectomy; ASA, American Society of Anesthesiologists; BMI, body mass index; RTH, radiotherapy; BC, breast cancer; PMRT, post-mastectomy radiotherapy; NAC, neo-adjuvant chemotherapy; N-RTH, neo-adjuvant radiotherapy.

## Data Availability

Not applicable.

## Abbreviations:

BC: breast cancer
IBR: immediate breast reconstruction
NSM: Nipple sparing mastectomy
SSM: Skin sparing mastectomy
BMI: Body mass index
ASA: American Society of Anesthesiologists
LPOS: Length of postoperative stay
OR: odds ratio
95% CI: 95% confidence interval
NAC: neoadjuvant chemotherapy
RTH: Radiotherapy
PMRT: Post mastectomy radiotherapy
N-RTH: Neo-adjuvant radiotherapy
SLNB: sentinel lymph node biopsy
ALND: axillary lymph node dissection
